# Eco-friendly consolidated process for co-production of xylooligosaccharides and fermentable sugars using self-providing xylonic acid as key pretreatment catalyst

**DOI:** 10.1186/s13068-019-1614-5

**Published:** 2019-11-18

**Authors:** Xin Zhou, Yong Xu

**Affiliations:** 1 Key Laboratory of Forestry Genetics & Biotechnology (Nanjing Forestry University), Ministry of Education, Nanjing, 210037 People’s Republic of China; 2 Jiangsu Co-Innovation Center of Efficient Processing and Utilization of Forest Resources, Nanjing Forestry University, Nanjing, 210037 People’s Republic of China; 3 College of Chemical Engineering, Nanjing Forestry University, No. 159 Longpan Road, Nanjing, 210037 People’s Republic of China

**Keywords:** Xylooligosaccharides, Xylonic acid, Xylose, Sugarcane bagasse, Enzymatic hydrolysis

## Abstract

**Background:**

Obtaining high-value products from lignocellulosic biomass is central for the realization of industrial biorefinery. Acid pretreatment has been reported to yield xylooligosaccharides (XOS) and improve enzymatic hydrolysis. Moreover, xylose, an inevitable byproduct, can be upgraded to xylonic acid (XA). The aim of this study was to valorize sugarcane bagasse (SB) by starting with XA pretreatment for XOS and glucose production within a multi-product biorefinery framework.

**Results:**

SB was primarily subjected to XA pretreatment to maximize the XOS yield by the response surface method (RSM). A maximum XOS yield of 44.5% was achieved by acid pretreatment using 0.64 M XA for 42 min at 154 °C. Furthermore, XA pretreatment can efficiently improve enzymatic digestibility, and achieved a 90.8% cellulose conversion. In addition, xylose, the inevitable byproduct of the acid-hydrolysis of xylan, can be completely converted to XA via bio-oxidation of *Gluconobacter oxydans* (*G. oxydans*). Subsequently, XA and XOS can be simultaneously separated by electrodialysis.

**Conclusions:**

XA pretreatment was explored and exhibited a promising ability to depolymerize xylan into XOS. Mass balance analysis showed that the maximum XOS and fermentable sugars yields reached 10.5 g and 30.9 g per 100 g raw SB, respectively. In summary, by concurrently producing XOS and fermentable sugars with high yields, SB was thus valorized as a promising feedstock of lignocellulosic biorefinery for value-added products.
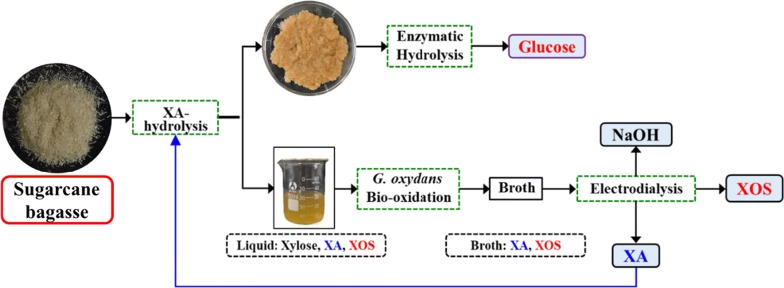

## Background

The global overconsumption of fossil fuels has driven the development of the lignocellulosic biorefinery concept, an industrial process that converts sustainably sourced lignocellulosic biomass into energy, chemicals, and fuels [1, 2]. A suitable candidate for such processing is sugarcane bagasse (SB), which is one of the most abundant types of lignocellulosic biomass in China and in other parts of the world [3]. SB is comprised of all biomass refuse after sugarcane harvest, and is usually discarded or burned at the field [4]. Both of these ongoing practices represent environmentally imprudent or even harmful usage of a readily available biomass resource [3–5]. The results of previous studies on the biorefining of SB indicated SB as well-suited for the production of value-added products especially due to its high polysaccharide content. Specifically, several sources of SB have been shown to consist of 40–50% cellulose and 20–30% hemicelluloses based on their dry mass [6, 7]. However, the natural structure of SB (and in general, of all lignocellulosic biomass) is strongly recalcitrant to enzymatic hydrolysis of polysaccharides (cellulose in particular). This characteristic thus necessitates the implementation of economical pretreatment that deconstructs the lignocellulosic entanglement without inducing damaging its constituents. To date, the pretreatment technologies that have received the most attention include hydrothermal, steam explosion, diluted acid, and diluted alkaline pretreatment [5, 8, 9].

Of particular note is that diluted acid pretreatment is not only beneficial for enzymatic hydrolysis, but it is also an effective mean to obtain desirable xylooligosaccharide (XOS) products from SB [10, 11]. This is because SB hemicelluloses are predominately composed of xylan. Here, XOS, from degradation of xylan, are short-chain oligosaccharides that are composed of 2–7 xylose molecules, tethered by β-(1,4) glycosidic linkages [12]. The most attractive biological feature of XOS is that it cannot be digested or absorbed by the human digestive system when consumed. Furthermore, it promotes the growth of beneficial intestinal bacteria. Therefore, XOS with a low degree of polymerization (DP), can be considered as key ingredients toward the improvement of gut health. Ingestion leads to a boost in calcium absorption, lowers cholesterol, improves the immune system, and reduces the risk of colon cancer [13–16]. In addition, the rapid growth of the functional food/feed in markets has boosted the demand for high-quality XOS, which the current market prices reflect as much as $22–50/kg [17–19]. Thus, it is beneficial to implement diluted acid pretreatment in a biorefinery process to optimize XOS production. Doing so would add an additional profit-generating product to the product portfolio of a biorefinery with the potential to achieve a high price.

Pretreatments with diluted acid use mineral or organic acid reagents, both of which have been reported to be capable of yielding XOS [20–22]. However, it has been suggested that mineral acids promote an increased extent of xylose and furfural production, which directly translates to lower XOS yields [20]. In contrast, organic acids tend to favor XOS generation, while also yielding additional benefits such as little furfural yield, lower corrosiveness and decreased generation of enzymatic hydrolysis inhibitors [11, 23, 24]. Various organic acids, such as acetic acid, oxalic acid, and gluconic acid, have already been explored as reagents for XOS production during acid pretreatment [25, 26]. Lin et al. [23] developed a microwave-induced hydrolysis of beechwood xylan with oxalic acid strategy to produce XOS, which achieved a yield of 39.31%. Zhang et al. [11] developed an acetic acid pretreatment method to produce XOS and improve enzymatic hydrolysis efficiency from corncob, and the acetic acid hydrolysis achieved XOS yield of 45.9%. In addition, Zhou et al. [24] found that gluconic acid exhibited a good ability to degrade SB xylan into XOS with a yield of 53.2%. It is important to note that this pretreatment technology effectively reduces biomass’ recalcitrance to enzymatic digestion [11, 27]. The key concern during diluted acid pretreatment for XOS production is to control how much xylose will be generated, which is an inevitable chemical reaction given the lability of glycosidic bonds in acidic media. Interestingly, previous reports suggested and successfully demonstrated that this xylose could be upgraded to xylonic acid (XA) and recovered as an additional product stream via electrodialysis [28, 29]. XA, with a structure similar to gluconic and derived from the oxidation of xylose, can also release H^+^ to depolymerize xylan [30]. In addition, it has been demonstrated that acidic pretreatment can efficiently weaken the hydrolyzed glycosidic bond of hemicellulose and lignin–hemicellulose bonds, which leads to sugar dissolution in the hemicellulose and to an increased porosity of the plant cell wall [6]. Overall, it is feasible to assume that XA can be used as an acid catalyst during diluted acid pretreatment. This study aspired to further optimize the acid pretreatment for obtain high value-added XOS that utilizes XA as key reagent. Thus, the influences of XA concentration, hydrolysis duration, and temperature on the composition of obtained hydrolysates were primarily assessed to maximize the XOS yield. Furthermore, the enzymatic hydrolysis efficiency of XA pretreated SB solids was also investigated. In summary, this work demonstrates an integrated biorefinery process that utilizes a reagent derived from biomass itself to produce XOS, a substrate that remains easily digestible by cellulolytic enzyme systems.

## Results and discussion

### Response surface method optimization of XOS yields

The main purpose of this study aspired to maximize the XOS yield due to the high value and the proportion of the soluble compounds (XOS) generally depends on the operation conditions. Here, temperature, acid concentration, and reaction time are crucial parameters in the hydrolysis of hemicelluloses. This is because each of these factors affects both hydrolysis rate and selectivity. Consequently, each of these three parameters was primarily optimized to achieve the highest XOS yield. RSM is an effective statistical procedure that uses a minimum set of experiments to determine the coefficients of a mathematical model as well as the optimum conditions. Thus, in the present study, SB samples were pretreated at different temperatures (130–170 °C) over a time range of 15–75 min with 3.0 g of SB and 30 mL XA solutions with different XA concentrations. Values of independent process variables were studied and the responses obtained from 13 different combinations of reaction conditions are shown in Table 1.

**Table 1:** Independent variables of the central composite design and results of response surface analysis [xylooligosaccharide (XOS) yields]

Variables			Responses
Reaction temperature (°C)	Reaction time (min)	XA concentration (mol/L)	XOS yields (%)
130	15	0.25	1.03
130	15	1	1.31
130	75	0.25	16.99
130	75	1	11.68
150	45	0.625	42.61
150	45	0	0.88
150	45	1	32.50
150	95	0.625	40.18
170	15	0.25	15.95
170	15	1	31.22
170	75	0.25	18.96
170	75	1	8.75
183	45	0.625	17.97

**Fig. 1:**
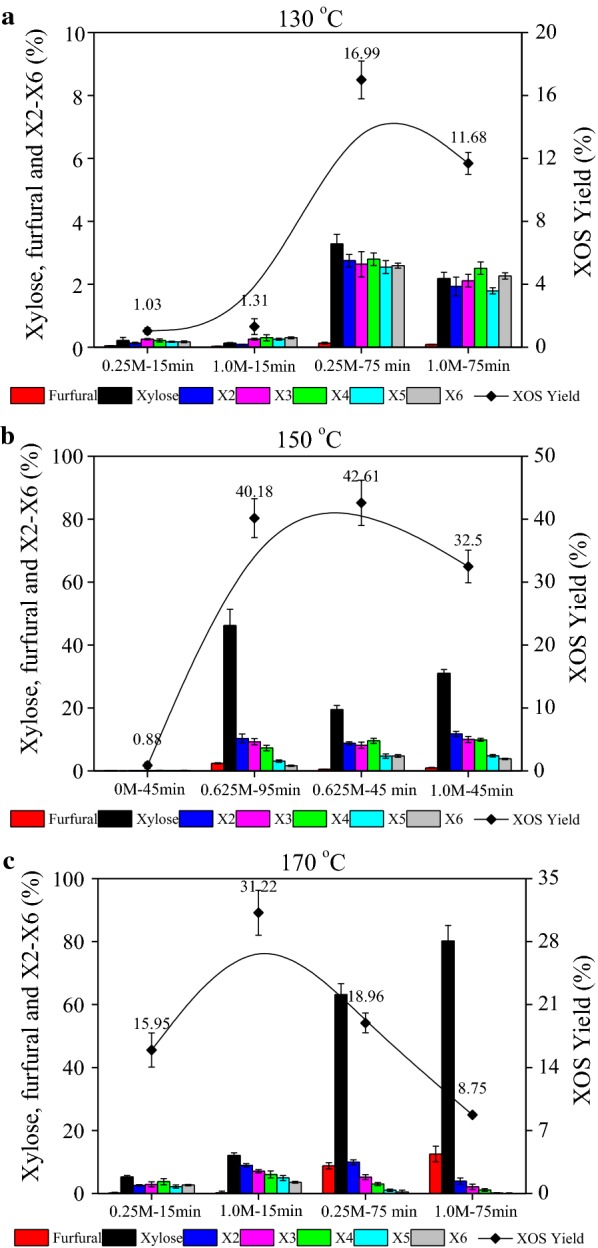
Yields of furfural, xylose, X2, X3, X4, X5, and X6 in hydrolysate produced from SB with different acid concentrations and times at **a** 130 °C, **b** 150 °C, and **c** 170 °C

After the XA hydrolysis of SB hemicelluloses was completed, the hydrolysis products in the hyrolysate of each sample were analyzed. Although XOS with DP larger than 6 could be generated, each sample reached up to DP 6, due to the lack of > DP6 of the standards, such as X7 and X8. Thus, X2–X6 were calculated for XOS yield, which is listed in Table 1. In addition, the relative contents of X2–X6, xylose, and furfural are shown in Fig. 1. According to the fit summary reports, the following regression equation represented the XOS yield from the experimental responses:

$$Y = - 859.7 + 10.65a_{1} + 1.92a_{2} + 127.75a_{3} - 0.01a_{1} a_{2} + 0.16a_{1} a_{3} - 0.34a_{2} a_{3} - 0.03a_{1}^{2} - 0.003a_{2}^{2} - 108.07a_{3}^{2}$$

**Fig. 2:**
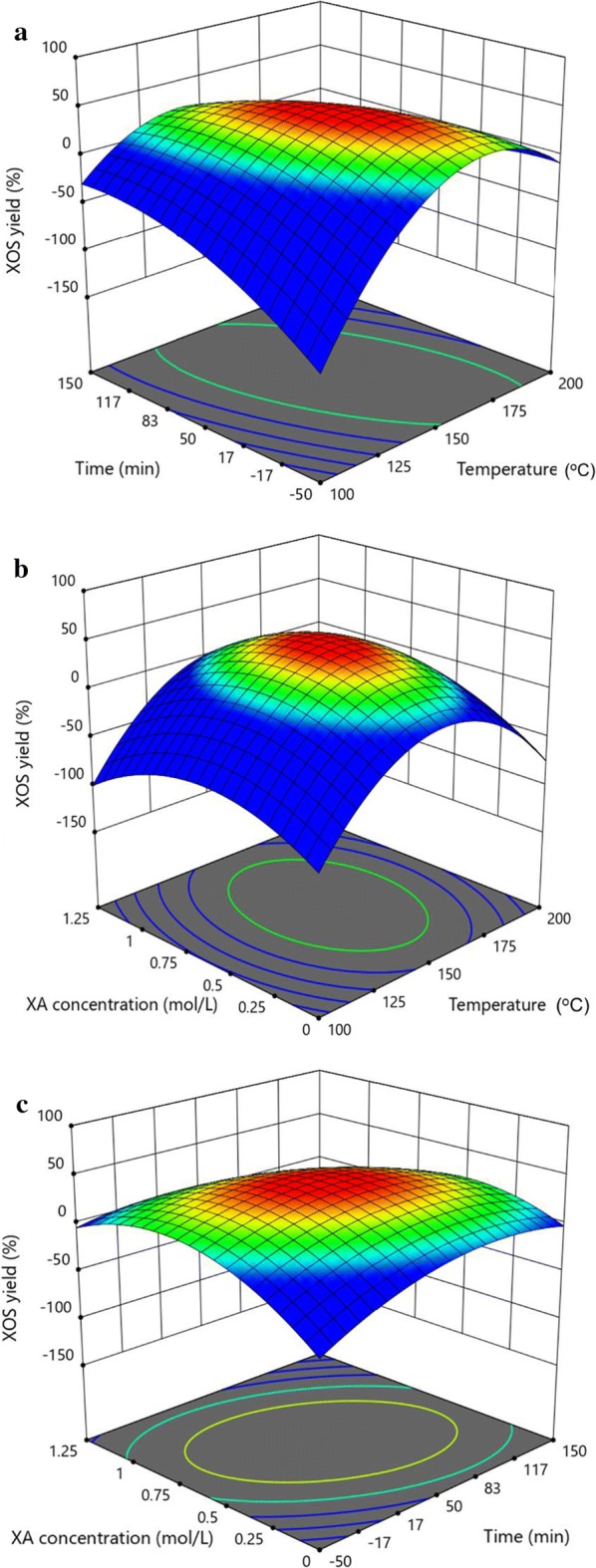
Response surface showing the effects of independent variables on XOS yields. **a** Reaction temperature (°C) and time (min); **b** XA concentration (mol/L) and reaction temperature (°C); **c** XA concentration (mol/L) and reaction time (min)

In the formula, *a*_1_, *a*_2_, and *a*_3_ represent temperature, hydrolysis time, and XA concentration, respectively. The determination coefficient *R*^2^ was used as correlation measure to test the goodness-of-fit of the regression equation, which is defined as the ratio of the explained variation to the total variation, and demonstrates the agreement between the observed and predicted results [31]. In the present study, the *R*^2^ for XOS yield was 0.974. The relatively high *R*^2^ values of the models indicate close agreement between the experimental results and the predicted values provided by the model. This similarity can also be verified by high correlations between the observed and predicted values (curves are supplied in Additional file 1). The results from analysis of variance yield a model’s *F*-value of 12.48 and a *P*-value of 0.031, indicating that the model’s quadratic terms accurately fitted the experimental data. Analysis of XOS yield showed that the *P*-values of *a*_1_, *a*_1_*a*_2_, *a*_1_^2^, and *a*_3_^2^ were < 0.05, indicating that the independent variable *a*_1_ and the quadratic terms of *a*_1_*a*_2_, *a*_1_^2^, and *a*_3_^2^ exerted significant effects on XOS yield. Two-dimensional contour plots and three-dimensional response surface plots were generated by Design-Expert software and is showed in Fig. 2a–c. The analysis results also demonstrated that the effects of the reaction temperature was more significant for XOS yield than that of hydrolysis time and acid concentration.

A relatively low temperature and short treatment time resulted in very low xylose and XOS yields. This is due to the time it takes to hydrolyze hemicelluloses into oligosaccharides. However, the XOS yield increased strongly from 1.3% (at 130 °C, 15 min, and 1.0 M) to 31.2% at elevated temperature (170 °C, 15 min, and 1.0 M). This was because the higher temperature accelerated the degradation of xylan-type hemicelluloses. Since the DP decreased with increasing acid concentration and hydrolysis time, the formation of monomers in the hydrolysate was inevitable. The results indicated that higher temperature and longer reaction times were conducive to the further degradation of XOS. With increased reaction temperature and acid concentration, X5, X6, and > X6 continued to be hydrolyzed into smaller oligosaccharides such as X2, X3, and X4. A higher level of X2 and X3 concentrations and lower amounts of X5 and X6 were observed at higher temperature with long reaction time. Although higher XOS yield (> 40%) also could be achieved by higher XA concentration with longer reaction time, more xylose and furfural were generated (150 °C, 95 min, and 0.625 M). As shown in Fig. 1a–c, the results indicated that the levels of both xylose and furfural increased during hydrolysis with increasing temperature and retention time.

The red zone in Fig. 2a–c shows the optimal condition for the production of XOS. The hydrolytic depolymerization of xylan-rich hemicelluloses proceeded most efficiently at 154 °C and 42 min with 0.64 M XA, with a predicted optimum XOS yield of 45.2%. The real contents of X2–X6 and xylose in hydrolysate by the optimized condition pretreatment were 3.22 g/L, 2.53 g/L, 2.37 g/L, 1.38 g/L, 0.96 g/L, and 7.11 g/L. Namely, the experimentally obtained XOS yield under this optimized condition was 44.5%. Clearly, the experimental values for XOS yields were found to be close to the predicted values obtained by the fitted model. The predicted optimum exactly matches the experimental optimum, thus verifying the accuracy of the established response surface. In addition, 0.28 g/L furfural was very low with this condition. Thus, condition using 154 °C and 42 min with 0.64 M XA is feasible for squeezing the maximum quantity of profit-generating products.

### Xylonic acid fermentation and separation

XOS are the prior target of this study since these are higher value-added products. The results presented above indicated that acid hydrolysis at high temperature can effectively depolymerize xylan-type hemicelluloses into XOS and achieve high yield. The results also proved useful to demonstrate XA as an effective catalyst for XOS production. However, xylose was also simultaneously generated during acid pretreatment. Under the optimum conditions for XOS production, 10.5 g/(100 g SB) XOS could be produced, and 7.1 g/(100 g SB) xylose was released into the hydrolysate. Moreover, approximately 102.1 g/L XA (as catalyst) still remained in the hydrolysate. In general, a commercial XOS product mainly consists of DP 2–6 with a purity of 70–95% [17]. Thus, both xylose and the catalyst (XA) need to be removed to produce XOS with sufficient purity. Previously, Cao et al. successfully purified sodium xylonate (XA·Na) broth via electrodialysis; however, the broth still contains residual sugar (xylose). Their results showed that XA, xylose, and NaOH could be separated simultaneously [29]. In addition, xylose can be converted into bio-oxidized XA by *G. oxydans* with a yield close to 100% [28, 32]. Overall, xylose can be converted to XA, which can then be separated by electrodialysis.

**Fig. 3:**
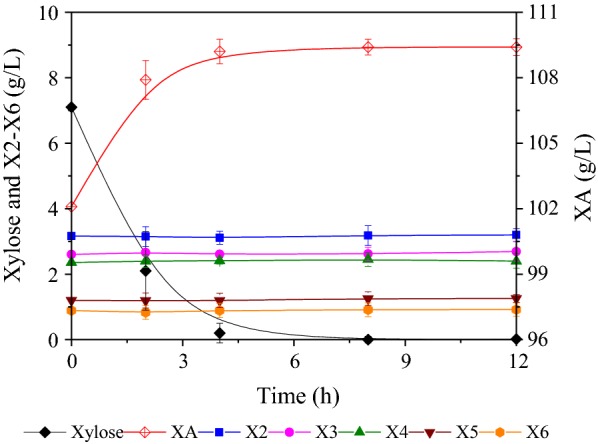
Xylose bioconversion by *Gluconobacter oxydans*

**Fig. 4:**
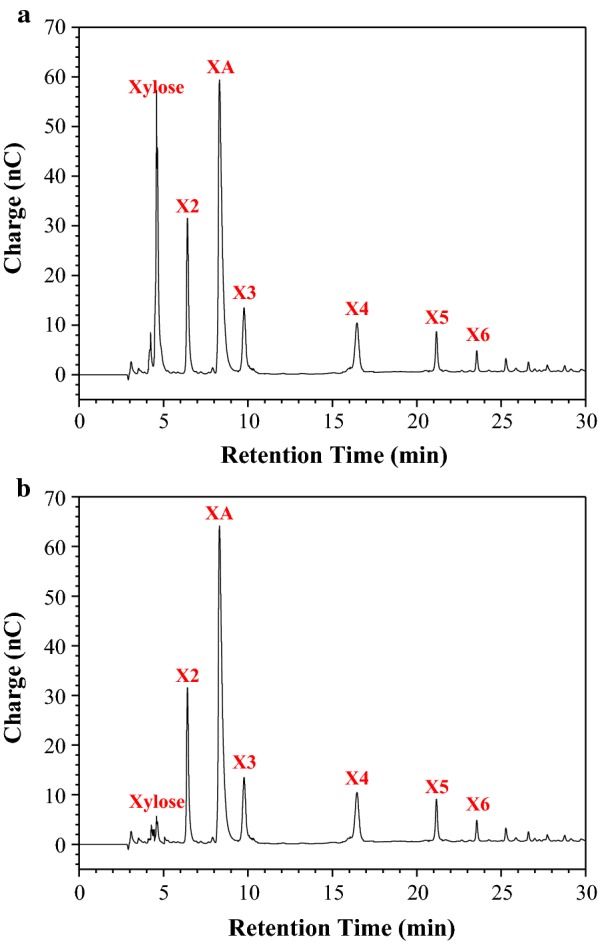
Chromatogram of high-performance anion exchange chromatography (HPAEC) analysis: **a** hydrolysate from xylonic acid (XA) pretreatment of sugarcane bagasse (SB); **b** hydrolysate after xylose bio-oxidation by *G. oxydans*

After XA pretreatment, the pH of pre-hydrolysate was adjusted to 6.0 using NaOH. pH adjustment is a requirement because a low pH inhibits the activity of *G. oxydans* cells. Next, the pre-hydrolysate was directly subjected to *G. oxydans* for the conversion of xylose into XA. During XA fermentation, NaOH was used to maintain the fermentation pH so that XA·Na remained in the hydrolysate. After 24 h of fermentation, approximately 109.1 g/L XA accumulated in the broth, with a corresponding yield of 95.1% (Fig. 3). Xylose could be completely and efficiently converted to XA by *G. oxydans*, even without addition of any further nutrients or inorganic salts to the hydrolysate. These observations also suggested that xylose was exclusively oxidized to XA without further catabolism. In addition, X2–X6 curves (Fig. 3) and chromatograms (Fig. 4a, b) confirmed that xylose was bio-converted into XA and XOS was not utilized by *G. oxydans*. This enabled the preservation of XOS and XA·Na for their downstream purification/separation. Thus, the XA-pre-hydrolysate was subjected to electrodialysis for XOS and XA separation and recovery. Electrodialysis was performed at a current density of 50 mA/cm^2^ and for about 30 min; 96.8% XA of the fermentation broth was recycled and close to 100% XOS were retained in the broth. In summary, electrodialysis with bipolar membranes is a very promising and prospective method for the simultaneous separation of ionic salts and for the recycling of sugar compounds for other uses.

### Enzymatic hydrolysis of pretreated solids

**Fig. 5:**
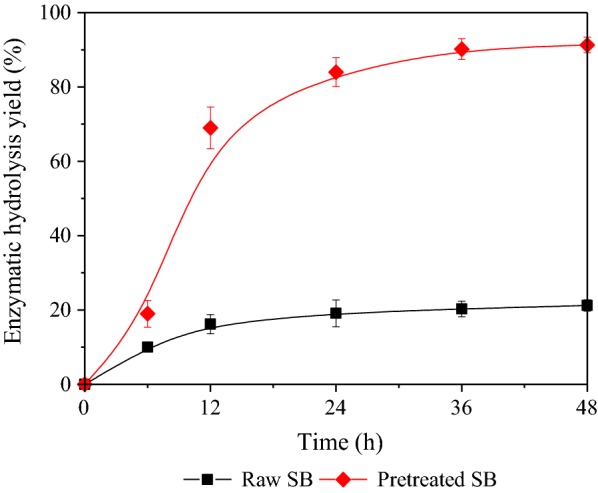
Yield of enzymatic hydrolysis after XA pretreatment

**Fig. 6:**
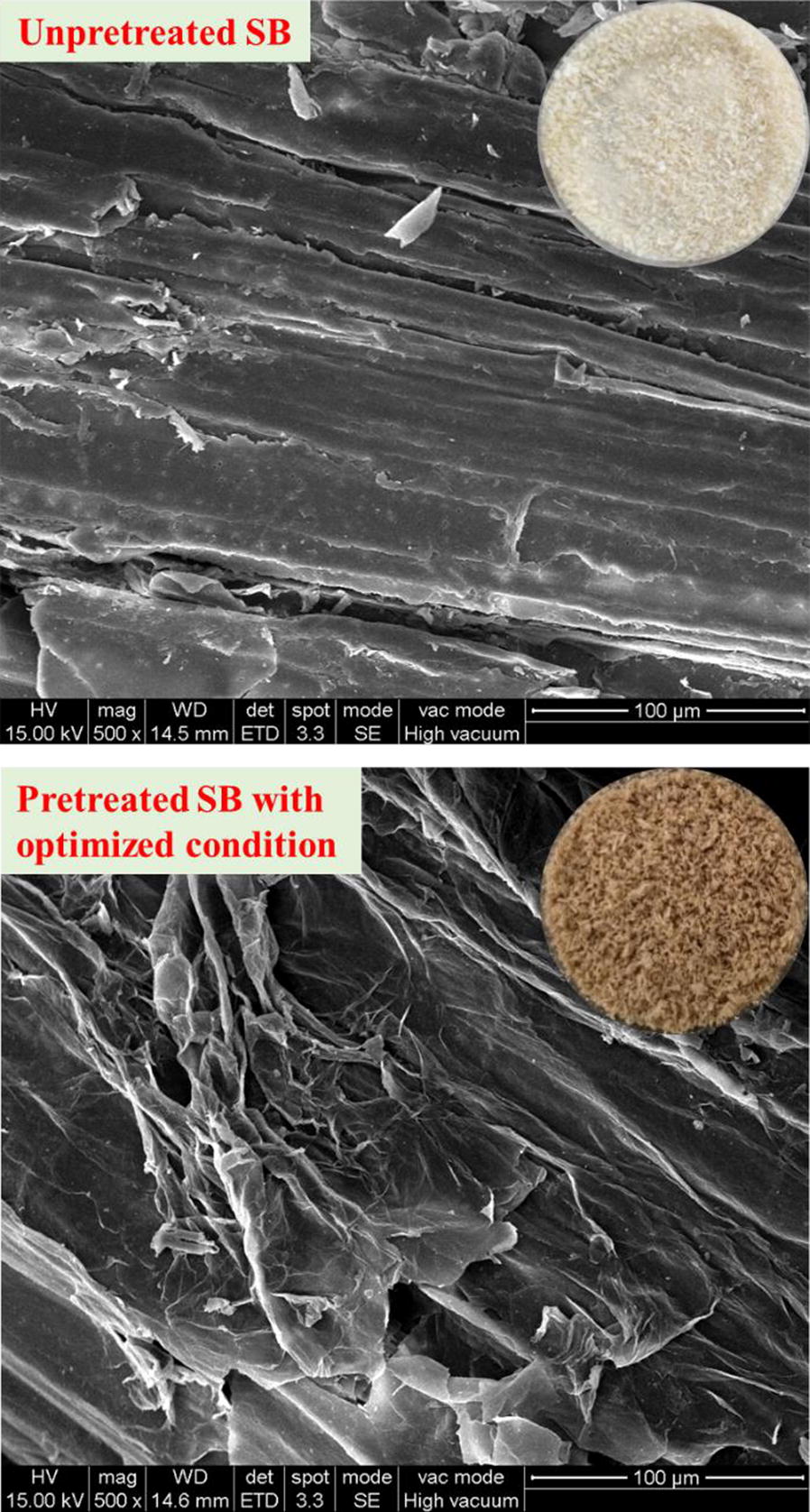
Scanning electron micrographs of treated and untreated SB

In the present study, XA (as an organic acid) was introduced to produce XOS, which also greatly changed the composition of SB. After XA pretreatment at RSM optimized conditions, the pretreated solids contained 54.6% glucan, 10.3% xylan, and 26.3% lignin. The hemicellulose fraction was mostly hydrolyzed during acid pretreatment, and higher cellulose and acid-insoluble lignin contents were recovered in the acid-pretreated solid residue. Furthermore, to investigate whether XA pretreatment can improve enzymatic hydrolysis, both raw and pretreated SB were subjected to enzymatic hydrolysis for 48 h at 5% w/v of the solid. Enzymatic hydrolysis was performed at 50 °C for 48 h and the enzymatic hydrolysis yield was expressed as the yield of glucose and cellobiose released into the enzymatic hydrolysate. Figure 5 showed that the XA pretreatment solid achieved a higher enzymatic hydrolysis yield. The enzymatic hydrolysis yield improved to 90.8% compared with the control without pretreatment (22.4%). It is noted that dilute acid pretreatments normally are capable of removing hemicellulosic fraction, but not lignin [33, 34]. Although the lignin cannot be effectively removed or degraded, another process occurring during acid pretreatment is lignin depolymerization and repolymerization through the formation of a common carbocation intermediate [35]. It has been proved that redistribution of lignin and degradation of hemicellulose jointly results in greatly altering the pore size distribution and the accessible surface area [36, 37]. As shown in Fig. 6, XA-pretreated SB featured a particularly higher quantity of newly exposed surfaces and generally rougher surface. All evidences indicated that XA pretreatment effectively improved the efficiency of enzymatic hydrolysis compared with the raw material.

**Fig. 7:**
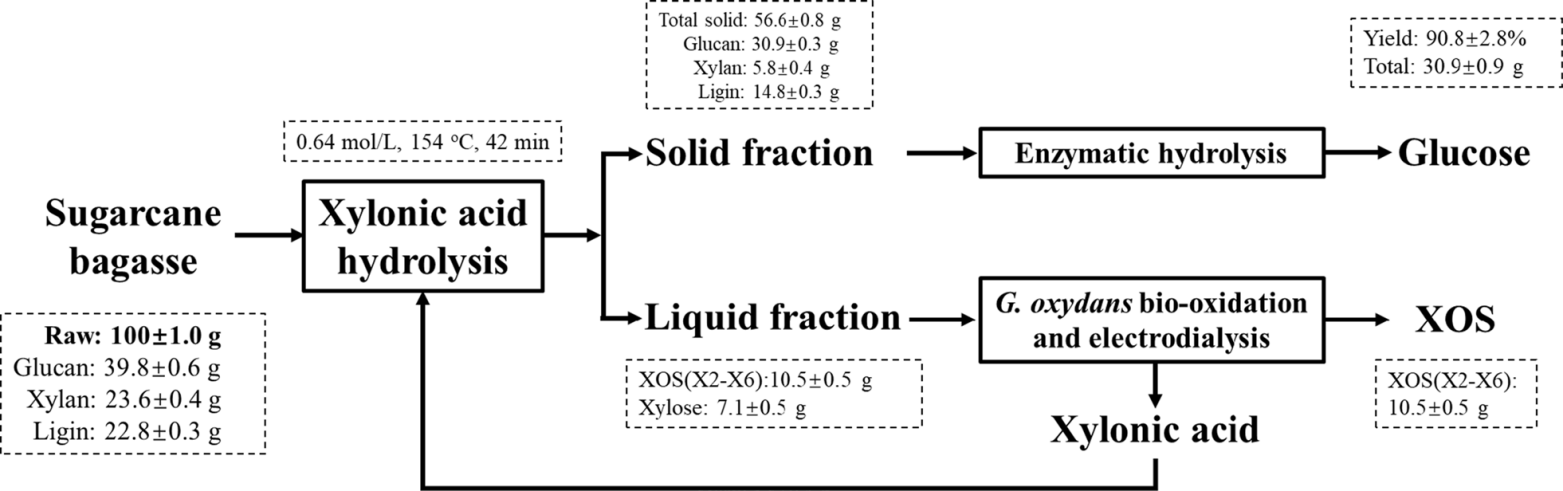
Mass balance for co-production of XOS and fermentable sugars

The mass balances were systematically analyzed during the co-production of glucose and XOS (Fig. 7). Using pretreated SB after XOS extraction as substrate and starting from 100 g of raw and dry SB, enzymatic hydrolysis obtained approximately 30.9 g of glucose and 10.5 g of XOS. The glucan and xylan recovery rates were 77.6% and 44.5%, respectively. These results clearly indicated pretreatment with XA as a promising option for the concurrent maximization of the economic value of SB by waste upgrading and the promotion of the current XOS and fermentable sugars co-production.

## Conclusions

This study used an agricultural waste (SB) as biorefinery feedstock for the production of high-value XOS and fermentable sugars. XA pretreatment was explored and exhibited great potential for the depolymerization of xylan into XOS. Mass balance analysis showed that the maximum yields of XOS and fermentable sugars reached 10.5 g and 30.9 g per 100 g of raw SB. In addition, xylose as byproduct from xylan hydrolysis can be converted into XA, which is able to be separated and recycled for running new acid pretreatment process. In summary, by concurrently producing XOS and glucose with XA pretreatment, SB was valorized as a promising feedstock for lignocellulosic biorefinery for the generation of value-added products.

## Methods

### Raw material and chemical composition analysis

SB was collected in Hainan Province, China, in the summer of 2019. In the laboratory, the material was broken into particles through 40 mesh. These particles were then oven-dried at 105 °C for 24 h until a constant weight was reached. The chemical composition (wt%) of SB was determined as follows: glucan 39.8%, xylan 23.6%, and lignin 22.8%.

### Experimental design for xylonic acid pre-hydrolysis

The hydrolysis parameters were optimized using RSM. A central composite design with three factors and three replicates at the center point was employed. Temperature (a1), hydrolysis time (a2), and XA concentration (a3) were chosen as independent variables. The ranges of these three independent variables and the center point values are listed in Table 1. In total, 13 experimental runs were conducted in three replicates with five center points, according to the Box–Behnken Design matrix. XOS yields were determined as the response variable (*Y*). The relationship between the independent variables and the response variable was calculated by a quadratic polynomial equation:

$$Y = a_{0} + \sum a_{i} a_{i} + \sum a_{ii} a_{i}^{2} + \sum a_{ij} a_{i} a_{j}$$

Here, *Y* represents the predicted XOS yields (%), *a*_0_ represents a constant term, *a* (*a*_*i*_ and *a*_*j*_) represent independent variables, and *a*_*i*_, *a*_*ii*_, and *a*_*ij*_ represent coefficients of linear, quadratic, and interaction parameters, respectively. The statistical software Design-Expert (Version 11.0) was used for a regression analysis of the experimental data and for response surface plots. Analysis of variance was used to estimate statistical parameters.

### Xylonic acid pretreatment of sugarcane bagasse

SB (3.0 ± 0.1 g) was mixed with 30 mL of different concentrations of XA solution (prepared via xylose bio-oxidation) in a 50-mL 316 stainless steel tube reactor (inner height × diameter is 80.0 × 28.0 mm, 5.0 mm wall) which also capped with 316 stainless steel cap. After loading, the sealed stainless steel tube reactor was immersed into preheated oil baths (dimethyl silicon oil) at different desired temperature, where it remained for varying durations depending on the utilized experimental design. The temperature of oil bath was controlled by the Parr PID controller. When the reaction finished, the stainless steel tube reactor immediately removed from the heater and cooled down using cold water, and then opened it. The resultant solid and liquid fractions were then separated and harvested by filtration. The separated liquid fraction was used to determine the XOS yields, while the solid fraction was subjected to component analysis and enzymatic hydrolysis.

### Xylose bioconversion

*Gluconobacter oxydans* (ATCC 621H) was used to convert xylose into the XA and maintained on plates (sorbitol 50 g/L, yeast extract 5 g/L, and agar 20 g/L) at 4 °C [29, 38]. The seed medium was prepared in a 500 mL Erlenmeyer flask, containing 100 mL medium (sorbitol 100 g/L and yeast extract 10 g/L), where it was cultured for 24 h at 220 rpm and 30 °C. Cell pellets of *G. oxydans* were harvested by centrifugation at 6000 rpm for 5 min. The pH of hydrolysate was adjusted to 6.0 by NaOH and then it was filtered by a 0.45-μm Millipore filter prior to inoculation. Fermentation assay was performed at 220 rpm and 30 °C with 1000 mL Erlenmeyer flask, containing 200 mL hydrolysate and 2 g/L *G. oxydans* cells (calculate as dry cell weight).

### Electrodialysis and separation of the fermented broth

A bipolar membrane electrodialyzer (GJBMED-90 × 210-5) from Xinke Co. (Liaoning, China) was used in this study and it consisted of a control unit (adjustable outputs of voltage 60 V and current 10 A). The parameters of electrodialysis are as follows. Single membrane effective area: 90 × 210 mm^2^; number of membrane stack repeat units: 15 pairs; electrode material: titanium-coated electrode plate; membrane material: polyphenylene oxide. [29]. In addition, electrodialysis equipment was composed of four chambers: acid, alkali, salt, and electrode chamber, and the working solution was online transported across the bipolar membrane to the acid chamber [39]. The electrodialysis stack was connected to a peristaltic pump. When starting the electrodialysis, sodium xylonate (Na·XA) in salt chamber was directly pumped into bipolar membrane electrodialysis stack. As a result, Na·XA was converted to XA with the protons produced from the water dissociation, which were transported into the compartment, while the Na^+^ was removed from the compartment. After ion exchange, XA accumulated in the acidic chamber, while the formed NaOH accumulated in the alkaline chamber. Finally, the aqueous solution (containing XOS) could be retained in salt chamber (The schematic of bipolar membrane electrodialysis was showed in Additional file 1) [29]. In addition, in the electrode chambers, 0.3 mol/L sodium sulfate solution was added to improve the conductivity and to reduce the resistance of the membrane stack.

### Enzymatic hydrolysis of pretreated sugarcane bagasse

Prior to enzymatic hydrolysis, solid residues from the XA pretreatment were washed with deionized water and were dried at room temperature until constant weight. Enzymatic hydrolysis was conducted in 150 mL screw-capped bottles at 50 °C, pH 4.8 (0.1 mol/L sodium acetate buffer), and 150 rpm with 5% solid loading and constant cellulases concentration (Cellic CTec2, Novozymes, NA, Franklinton, USA) of 15 FPU/g glucan. After enzymatic hydrolysis, the rendered enzymatic hydrolysate was collected by centrifugation.

### Analytical methods

The chemical composition of SB (cellulose, hemicelluloses, and lignin) was determined using a standard protocol provided by the National Renewable Energy Laboratory [40]. Carbohydrate contents (glucan and xylan) and lignin (acid-insoluble lignin and acid-soluble lignin) were analyzed for the raw material and the pretreated samples following two-step sulfuric acid pretreatment. Briefly, milled samples (20–80 meshes) were first hydrolyzed by 72% (w/w) sulfuric acid at 30 °C water bath for 1 h with frequent mixing. Then, the slurry was diluted to a concentration of 4% (w/w) sulfuric acid by adding deionized water and hydrolyzed at 121 °C for 1 h. The autoclaved slurry was cooled and filtered by filtering crucibles. The separated liquid fraction was used for analysis carbohydrates and acid-soluble lignin. The carbohydrates were analyzed by high performance liquid chromatography (HPLC) (Agilent 1260, USA) equipped with an Aminex Bio-Rad HPX-87H column (Bio-Rad Laboratories, USA). HPLC was performed at 50 °C, with 0.005 M sulfuric acid as eluent at a flow rate of 0.6 mL/min. The acid-soluble lignin was determined at 240 nm by UV spectrophotometer (UV-1800, Shimadzu, Japan). The separated solid fraction was dried at 105 °C oven until constant weight. After record the weight, the dry solid was heated at 575 °C muffle oven for 4 h to calculate the insoluble lignin.

Microscopic images of the raw and pretreated SB were captured by a scanning electron microscope (FEI Quanta 400, Hitachi, Japan), which was operated at a voltage of 15 kV. Prior to observation, all samples were sputter-coated with gold. SEM photomicrographs were taken at a magnification of 500×.

XA, xylose, xylobiose (X2), xylotriose (X3), xylotetraose (X4), xylopentaose (X5), and xylohexaose (X6) were analyzed by high-performance anion exchange chromatography (HPAEC) (Dionex ICS-5000, ThermoFisher, USA) coupled with a CarboPac™ PA200 column (ThermoFisher, USA) [41]. Glucose, cellobiose, and furfural were measured according to the HPLC method described above.

XOS, enzymatic hydrolysis [42], and XA yield were calculated as follows:

$${\text{XOS}}\;{\text{yield }}(\% ) = \frac{{({\text{X}}2 + {\text{X}}3 + {\text{X}}4 + {\text{X}}5 + {\text{X}}6)\;{\text{in}}\;{\text{hydrolysate}}\left( {\text{g}} \right)}}{{{\text{Initial}}\;{\text{xylan}}\;{\text{content}}\;{\text{in}}\;{\text{the}}\;{\text{raw}}\;{\text{material}}\left( {\text{g}} \right)}} \times 100\%$$

$${\text{Enzymatic}}\;{\text{hydrolysis}}\;{\text{yield}}\left( \% \right) = \frac{{({\text{Glucose}} + 1.053 \times {\text{cellobiose}})\;{\text{in}}\;{\text{enzymatic}}\;{\text{hydrolysate}}\left( {\text{g}} \right)}}{{1.111 \times {\text{Glucan}}\;{\text{content}}\;{\text{after}}\;{\text{XA}}\;{\text{hydrolysis}}\left( {\text{g}} \right)}} \times 100\%$$

$${\text{XA}}\;yield\left( \% \right) = \frac{{{\text{XA}}\;{\text{concentration}} \left( {\text{g/L}} \right) \times 0.918 }}{{{\text{Xylose}}\;{\text{in}}\;{\text{hydrolysate }}\left( {\text{g/L}} \right)}} \times 100\%$$

## Supplementary information

**Additional file 1.** Actual vs. predicted xylooligosaccharide (XOS) yields from XA hydrolysis of SB. Schematic of bipolar membrane electrodialysis.

## Data Availability

All data generated or analyzed during this study are included in this published article.

## Abbreviations

XOS: Xylooligosaccharides
XA: Xylonic acid
SB: Sugarcane bagasse
XA·Na: Sodium xylonate
DP: Degrees of polymerization
X2: Xylobiose
X3: Xylotriose
X4: Xylotetraose
X5: Xylopentaose
X6: Xylohexose
X7: Xyloheptaose
X8: Xylooctaose
RSM: Response surface method
HPLC: High-performance liquid chromatography
HPAEC: High-performance anion exchange chromatography
