# Supplementary material for: Eco-friendly consolidated process for co-production of xylooligosaccharides and fermentable sugars using self-providing xylonic acid as key pretreatment catalyst
Source: Biotechnol Biofuels. 2019 Nov 18;12:272. doi: 10.1186/s13068-019-1614-5 (PMC6859624; doi:10.1186/s13068-019-1614-5)
Supplement: Supplementary file 1 — Additional file 1. Actual vs. predicted xylooligosaccharide (XOS) yields from XA hydrolysis of SB. Schematic of bipolar membrane electrodialysis. [file 13068_2019_1614_MOESM1_ESM.docx]

**Additional material**

Actual vs. predicted xylooligosaccharide (XOS) yields from XA hydrolysis of SB.

**
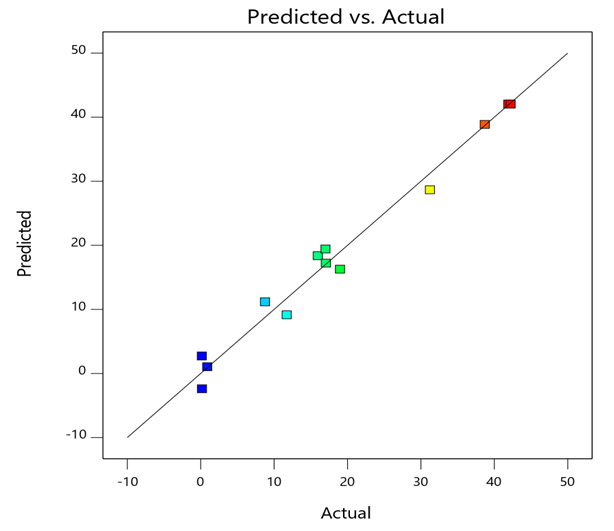
**

Schematic of bipolar membrane electrodialysis
